# Supplementary material for: SpyMask enables combinatorial assembly of bispecific binders
Source: Nat Commun. 2024 Mar 16;15:2403. doi: 10.1038/s41467-024-46599-9 (PMC10944524; doi:10.1038/s41467-024-46599-9)
Supplement: Supplementary file 1 — Supplementary Information [file 41467_2024_46599_MOESM1_ESM.pdf]

Supplementary Figures

SpyMask Enables Combinatorial Assembly of Bispecific Binders

Claudia L. Driscoll[1,2], Anthony H. Keeble[1,2], and Mark R. Howarth[1,2]*
[1]Department of Biochemistry, University of Oxford, South Parks Road, Oxford, OX1 3QU, UK.
[2]Department of Pharmacology, University of Cambridge, Tennis Court Road, Cambridge, CB2 1PD, UK.

*Corresponding author and Lead Contact:
Mark Howarth,
Department of Pharmacology,
University of Cambridge,
Tennis Court Road, Cambridge,
CB2 1PD, UK
E-mail: mh2186@cam.ac.uk

SpyTag003

RGVPHIVMVDAYKRYK

Masked SpyCatcher003

MSYYHHHHHHDYDIPTTGAMVTTLSGLSGEQGPSGDMTTEEDSATHIKFSKRDEDGRELAGATMELRDSSGKTISTWIS
DGHVKDFYLYPGKYTFVETAAPDGYEVATPIEFTVNEDGQVTVDGEATEGDAGSSGSENLYFQGGGSG**RGVPHIVMVAA
YKRYK****

DoubleCatcher

MSYYHHHHHHDYDIPTTENLYFQGGAMVTTLSGLSGEQGPSGDMTTEEDSATHIKFSKRDEDGRELAGATMELRDSSGK
TISTWISDGHVKDFYLYPGKYTFVETAAPDGYEVATPIEFTVNEDGQVTVDGEATEGDAHTGSGGSGGSGVTTLSGLSG
EQGPSGDMTTEEDSATHIKFSKRDEDGRELAGATMELRDSSGKTISTWISDGHVKDFYLYPGKYTFVETAAPDGYEVAT
PIEFTVNEDGQVTVDGEATEGDAGSSGSENLYFQGGGSG**RGVPHIVMVAAYKRYK***

DoubleCatcher H-Lock

MSYYHHHHHHDYDIPTTENLYFQGGAMVTTLSGLSGEQGPSGDMTTEEDSATHIKFSKRDEDGRELAGATMELRDSSGK
TISTWISDGHVKDFYLYPGKYTFVETAAPDGYEVATPIEFTVNEDGQVTVDGEATEGDAHTGSPANLKALEAQKQKEQR
QAAEELANAKKLKEQLEKGSVTTLSGLSGEQGPSGDMTTEEDSATHIKFSKRDEDGRELAGATMELRDSSGKTISTWIS
DGHVKDFYLYPGKYTFVETAAPDGYEVATPIEFTVNEDGQVTVDGEATEGDAGSSGSENLYFQGGGSG**RGVPHIVMVAA
YKRYK***

DoubleCatcher α-Lock

MSYYHHHHHHDYDIPTTENLYFQGGAMVTTLSGLSGEQGPSGDMTTEEDSATHIKFSKRDEDGRELAGATMELRDSCGK
TISTWISDGHVKDFYLYPGKYTFVETAAPDGYEVATPIEFTVNEDGQVTVDGEATEGDAHTGSGGSGGSGVTTLSGLSG
EQGPSGDMTTEEDSATHIKFSKRDEDGRELAGATMELRDSCGKTISTWISDGHVKDFYLYPGKYTFVETAAPDGYEVAT
PIEFTVNEDGQVTVDGEATEGDAGSSGSENLYFQGGGSG**RGVPHIVMVAAYKRYK***

DoubleCatcher β-Lock

MSYYHHHHHHDYDIPTTENLYFQGGAMVTTCSGLSGEQGPSGDMTTEEDSATHIKFSKRDEDGRELAGATMELRDSSGK
TISTWISDGHVKDFYLYPGKYTFVETAAPDGYEVATPIEFTVNEDGQVTVDGEATEGDAHTGSGGSGGSGVTTLSGLSG
EQGPCGDMTTEEDSATHIKFSKRDEDGRELAGATMELRDSSGKTISTWISDGHVKDFYLYPGKYTFVETAAPDGYEVAT
PIEFTVNEDGQVTVDGEATEGDAGSSGSENLYFQGGGSG**RGVPHIVMVAAYKRYK***

DoubleCatcher γ-Lock

MSYYHHHHHHDYDIPTTENLYFQGGAMVTTLSGLSGEQCPSGDMTTEEDSATHIKFSKRDEDGRELAGATMELRDSSGK
TISTWISDGHVKDFYLYPGKYTFVETAAPDGYEVATPIEFTVNEDGQVTVDGEATEGDAHTGSGGSGGSGVTTLSGLSG
EQGPSGDMTTEEDSATHIKFSKRDEDGRELAGATMELRDSSCKTISTWISDGHVKDFYLYPGKYTFVETAAPDGYEVAT
PIEFTVNEDGQVTVDGEATEGDAGSSGSENLYFQGGGSG**RGVPHIVMVAAYKRYK***

DoubleCatcher δ-Lock

MSYYHHHHHHDYDIPTTENLYFQGGAMVTTLSGLSGEQGPSGDMTTEEDSATHIKFSKRDEDGRELAGATMELRDSSGK
TISTWISDGHVKDFYLCPGKYTFVETAAPDGYEVATPIEFTVNEDGQVTVDGEATEGDAHTGSGGSGGSGVTTLSGLSG
EQGPSGDMTTEEDSATHIKFSKRDEDGRELAGATMELRDSSGKTISTWISDGHVKDFYLCPGKYTFVETAAPDGYEVAT
PIEFTVNEDGQVTVDGEATEGDAGSSGSENLYFQGGGSG**RGVPHIVMVAAYKRYK***

DoubleCatcher ε-Lock

MSYYHHHHHHDYDIPTTENLYFQGGAMVTTLSGLSGEQGPSGDMTTEEDSATHIKFSKRDEDGRELAGATMELRDSSGC
TISTWISDGHVKDFYLYPGKYTFVETAAPDGYEVATPIEFTVNEDGQVTVDGEATEGDAHTGSGGSGGSGVTTLSGLSG
EQGPSGDMTTEEDSATHIKFSKRDEDGRELAGATMELRDSSGKTISTWISDGCVKDFYLYPGKYTFVETAAPDGYEVAT
PIEFTVNEDGQVTVDGEATEGDAGSSGSENLYFQGGGSG**RGVPHIVMVAAYKRYK***

Supplementary Figure 1. **Amino acid sequences of SpyTag003, Masked SpyCatcher003, and DoubleCatcher variants.** The His$_6$-tag is shown with gray shading, TEV protease cleavage site with green shading, GSG spacer with cyan shading, cysteines in red, SpyTag003DA mask in bold, and the stop codon with *.

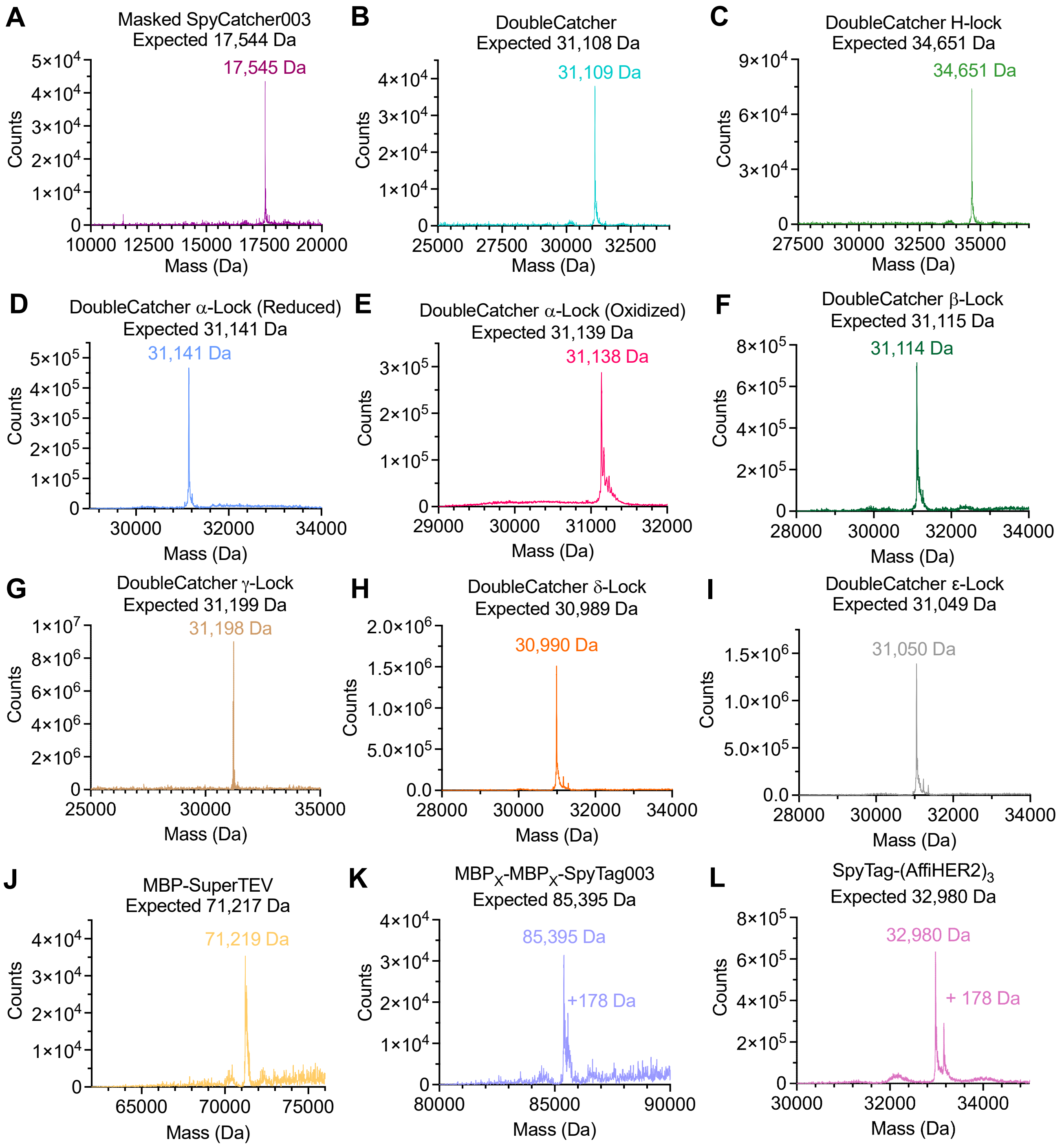

Supplementary Figure 2. **Mass spectrometry of building blocks.** Electrospray Ionization Mass Spectrometry on Masked SpyCatcher, DoubleCatcher variants, and Tagged components used for the assembly of bispecifics *(n* = 1 experiment per sample). The observed mass is indicated above the main peak. The expected mass was calculated from ExPASy ProtParam. The minor +178 Da peak relates to gluconoylation, which is a common post-translational modification for proteins overexpressed in *E. coli* BL21 (Geoghegan et al., 1999). Source data are provided as a Source Data file.

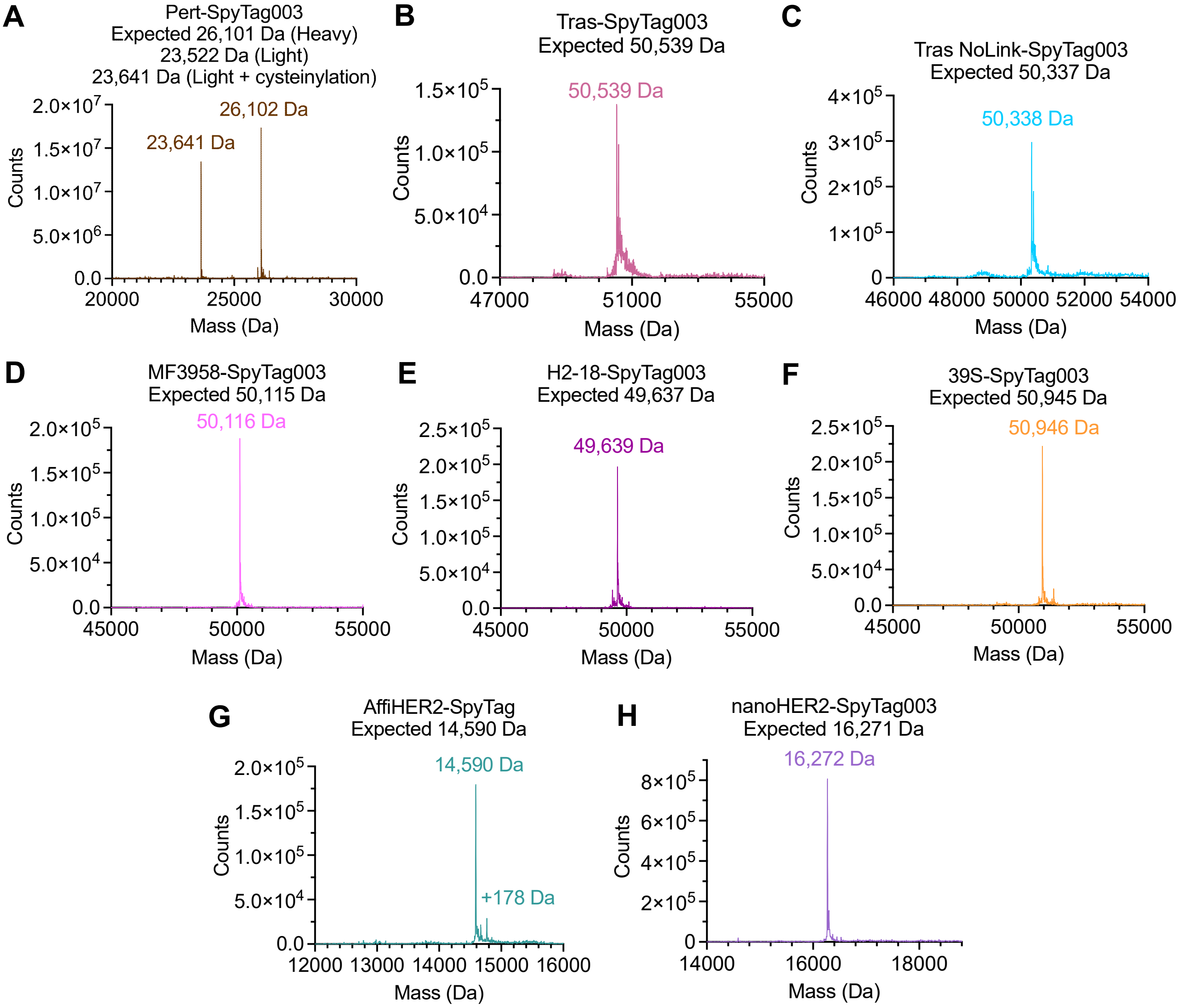

Supplementary Figure 3. **Mass spectrometry of anti-HER2 binders.** Electrospray Ionization Mass Spectrometry of Tagged anti-HER2 Fabs, an affibody, and a nanobody, as used to assemble the matrix of bispecifics bispecifics (*n* = 1 experiment per sample). The observed mass is indicated above the main peak. Since the hinge region is omitted from the Pert-SpyTag003 heavy chain sequence, there is no interchain disulfide bond in the Pert Fab. The observed +119 Da mass of Pert-SpyTag003 light chain relates to cysteinylation, as previously observed on human IgG1 kappa light chains (Lim et al., 2001). The minor +178 Da peak relates to gluconoylation (Geoghegan et al., 1999). The expected mass was calculated from ExPASy ProtParam. Source data are provided as a Source Data file.

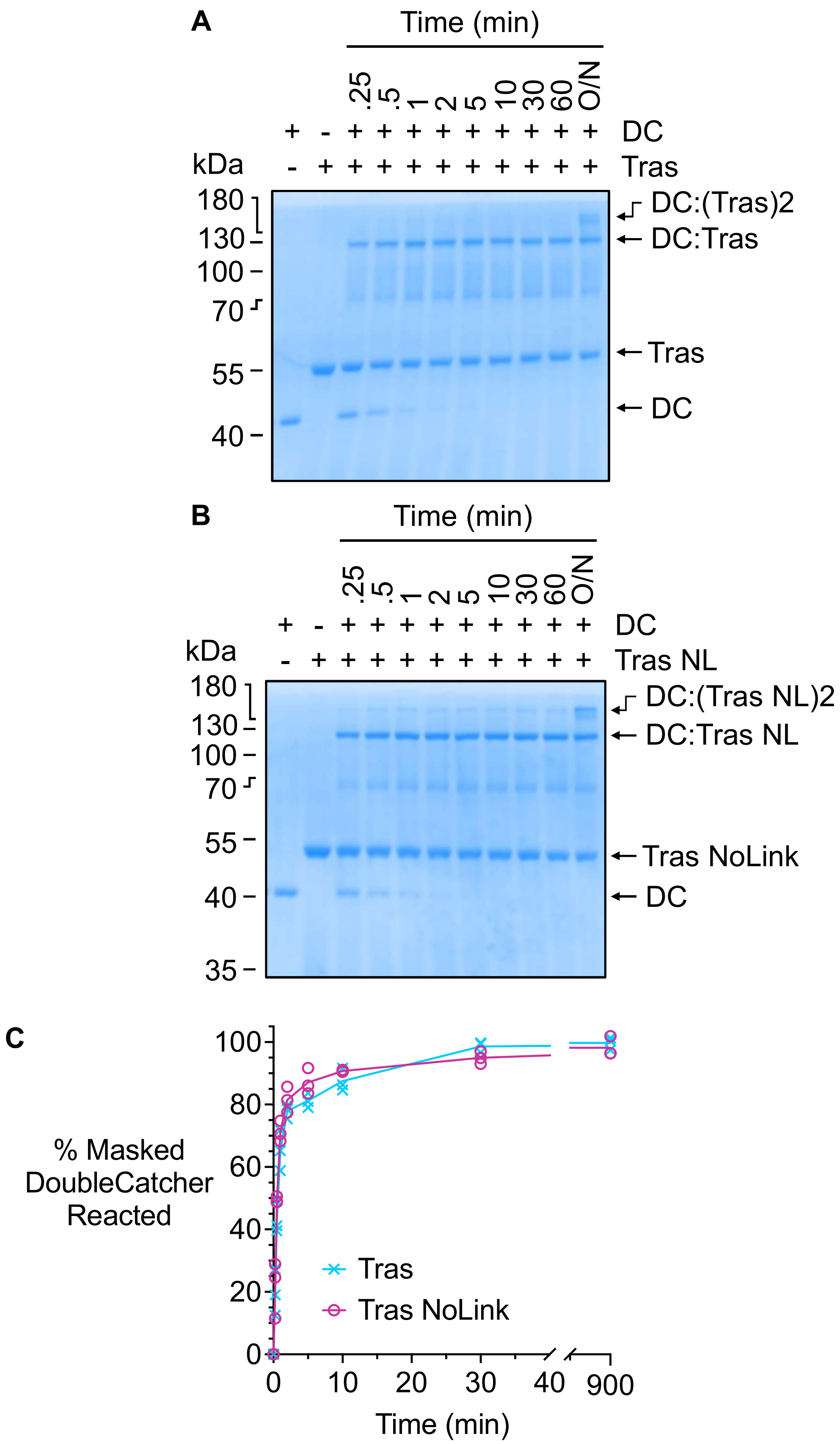

Supplementary Figure 4. **Removing the linker between Tras Fab and SpyTag003 does not affect reactivity with DoubleCatcher.** The tripeptide linker connecting Tras Fab (Tras) and SpyTag003 was removed to generate Tras NoLink (Tras NL), to reduce the flexibility between the DoubleCatcher and the binder. (**A**) Time-course of reaction between 2.5 µM DoubleCatcher (DC) and 5 µM Tras in PBS pH 7.4 at 37 °C for the indicated time, analyzed by SDS-PAGE/Commassie *(n* = 1 gel per triplicate time-course reaction). (**B**) As in (A) with Tras NL *(n* = 1 gel per triplicate time-course reaction). (**C**) Quantification of reactivity of Tras or Tras NoLink for DoubleCatcher. Each datapoint is shown (*n* = 3 independent reactions), with the line connecting the mean. Source data are provided as a Source Data file.

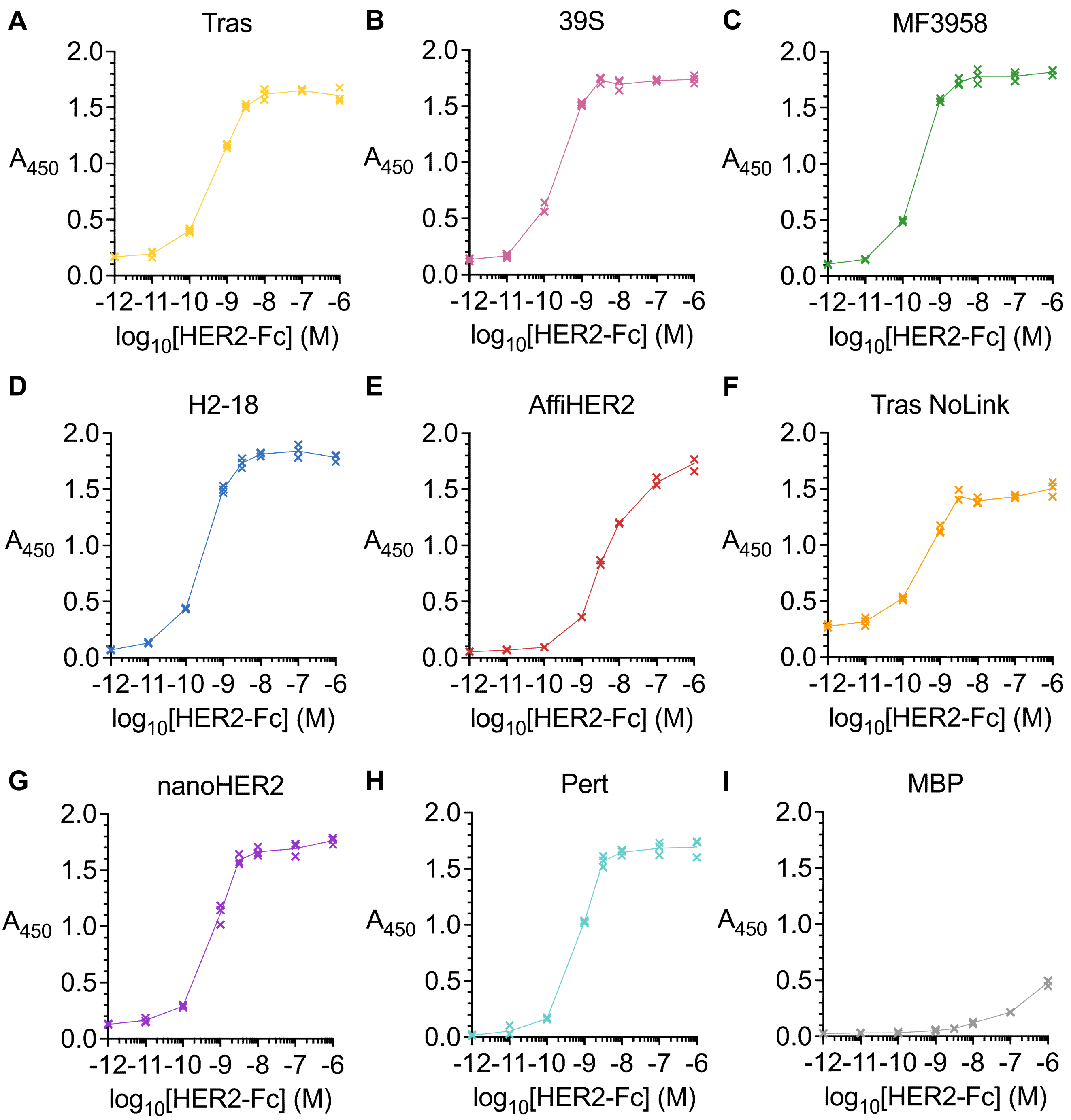

Supplementary Figure 5. **Validation of binder panel interaction with HER2 by ELISA.** The binding activity of each anti-HER2 binder used in this study to recombinant HER2 ECD-Fc was measured by ELISA. The binder was coupled to the well and then HER2 ECD-Fc was added at the indicated concentration, before detection with anti-Fc HRP. MBP was an irrelevant protein to serve as a negative control. Each triplicate data point is shown, with the line connecting the mean. *(n* = 3 technical replicates; ELISA experiments were repeated twice with similar results). Source data are provided as a Source Data file.

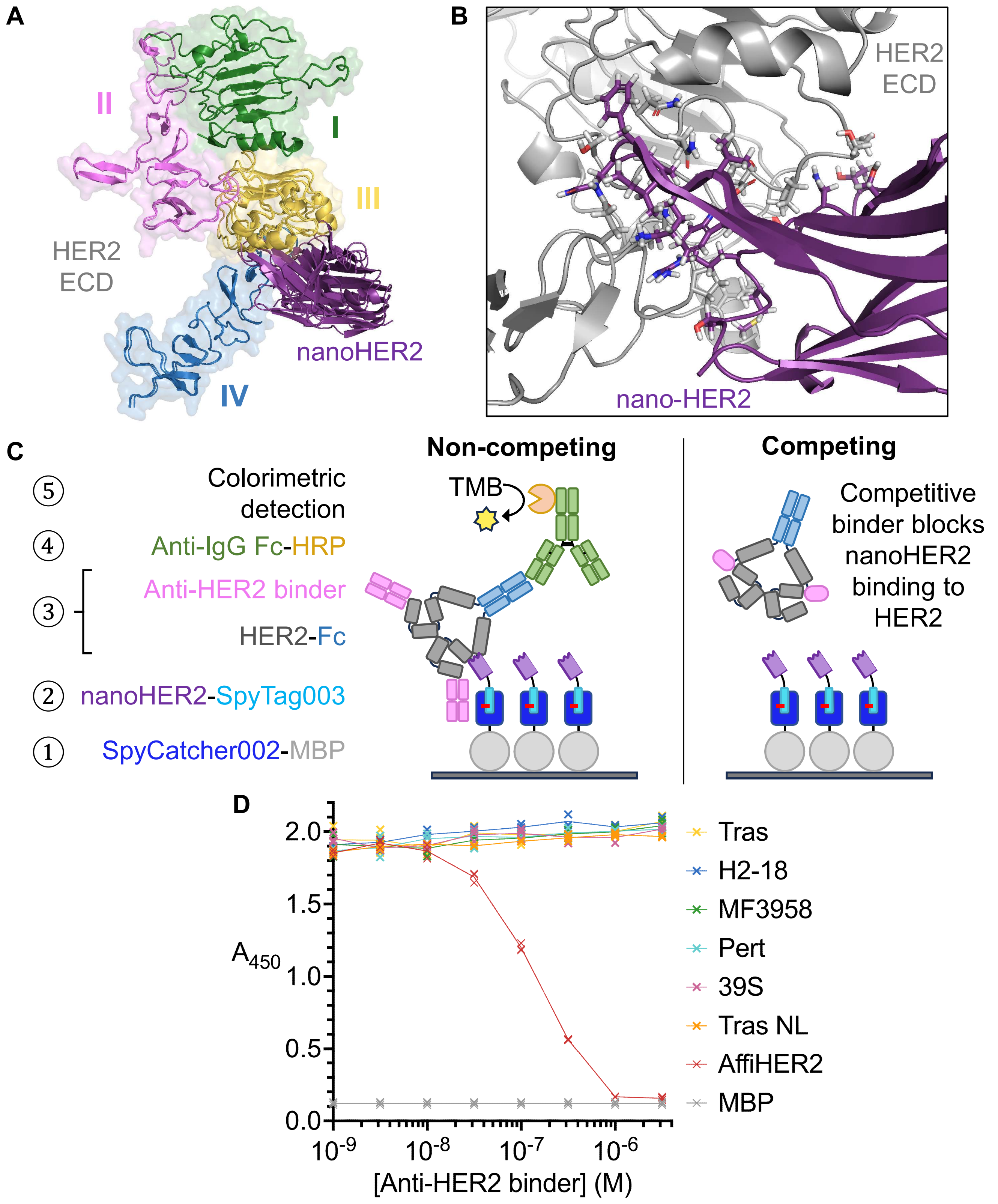

Supplementary Figure 6. **Elucidation of nanoHER2 binding epitope on HER2 from computation and competitive ELISA.** (**A**) AlphaFold-multimer-predicted docking of nanoHER2 with HER2 ECD. The three structures which predict ≥ 2 nanobody loops contributing to the interaction interface are overlaid. Subdomains of HER2's ECD are colored with surface representation and cartoon, with nanoHER2 represented in purple cartoon format. (**B**) The binding interface between the highest ranked nanoHER2 AlphaFold2 docking structure from (A) with HER2 ECD. Residues at the interface are shown in stick format. (**C**) Schematic of the competition ELISA for elucidation of the HER2 epitope bound by nanoHER2. Numbers indicate the order of addition to the well. HER2 ECD-Fc pre-incubated with non-competing binders can interact with nanoHER2, allowing colorimetric detection by anti-human IgG Fc-HRP and TMB substrate ('Non-competing' panel, left). HER2 ECD-Fc pre-incubated with a competing binder will not be bound by nanoHER2 and will not be detected ('Competing' panel, right). (**D**) Competition ELISA on nanoHER2 with HER2 ECD-Fc pre-incubated with the indicated amounts of each anti-HER2 binder. Triplicate points are shown with the line connecting the mean. MBP is a negative control where wells were reacted with SpyTag003-MBP instead of nanoHER2-SpyTag003. (*n* = 3 technical replicates, but ELISA experiments were repeated twice with similar results). Source data are provided as a Source Data file.

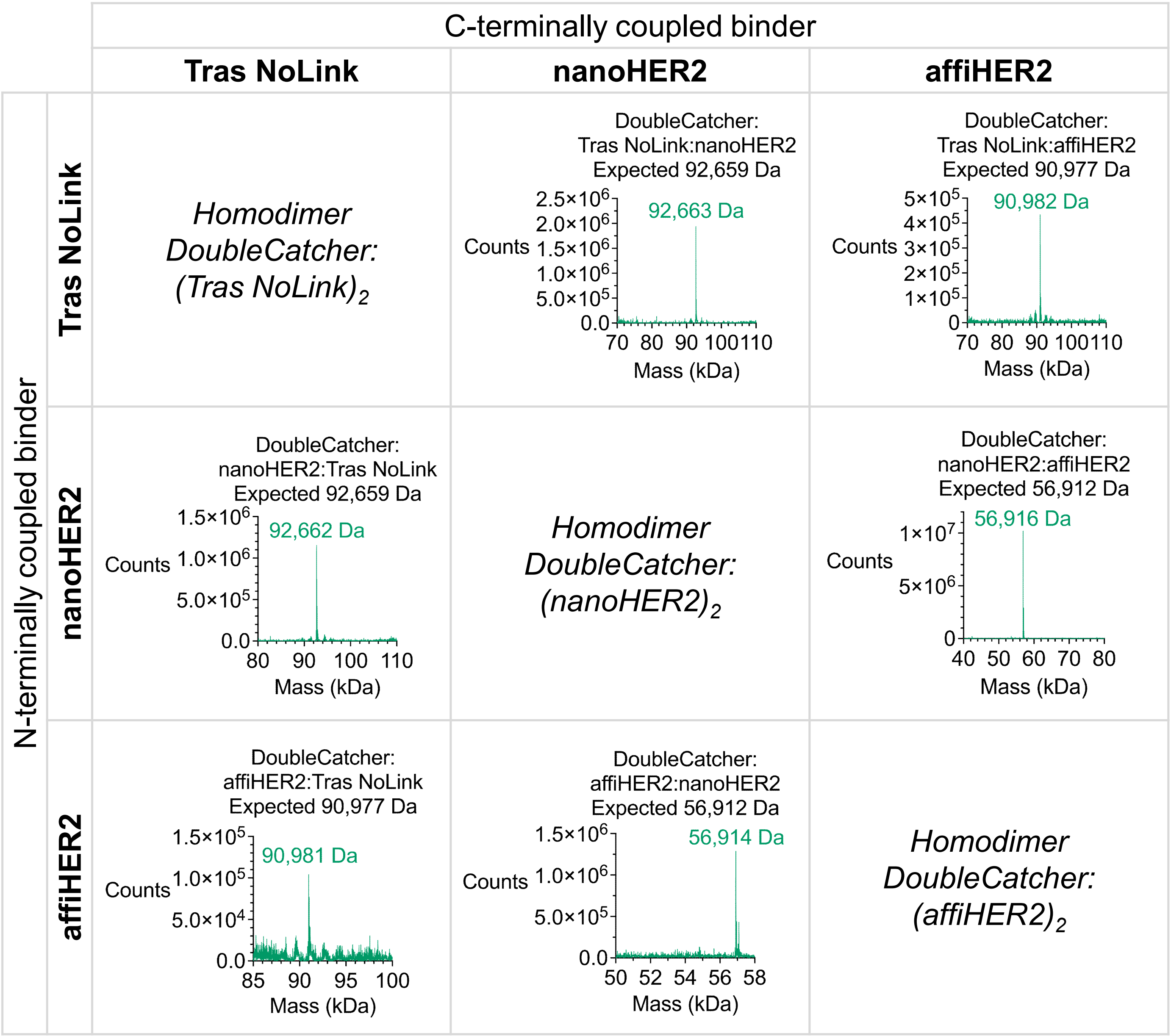

Supplementary Figure 7. **Heterodimerization by DoubleCatcher was validated by mass spectrometry.** Heterodimerization of a subset of anti-HER2 binders (Tras NoLink Fab, nanoHER2 nanobody, and affiHER2 affibody) in each possible combination was performed using DoubleCatcher to generate bispecific binders. The identity of the heterodimer species of the expected molecular weight was confirmed by Electrospray Ionization Mass Spectrometry, presented within a grid *(n* = 1 experiment per sample, but results were confirmed once with independently-assembled bispecific binders). Rows indicate the binder at the N-terminus of DoubleCatcher within the bispecific, while columns indicate the binder at the C-terminus. Source data are provided as a Source Data file.

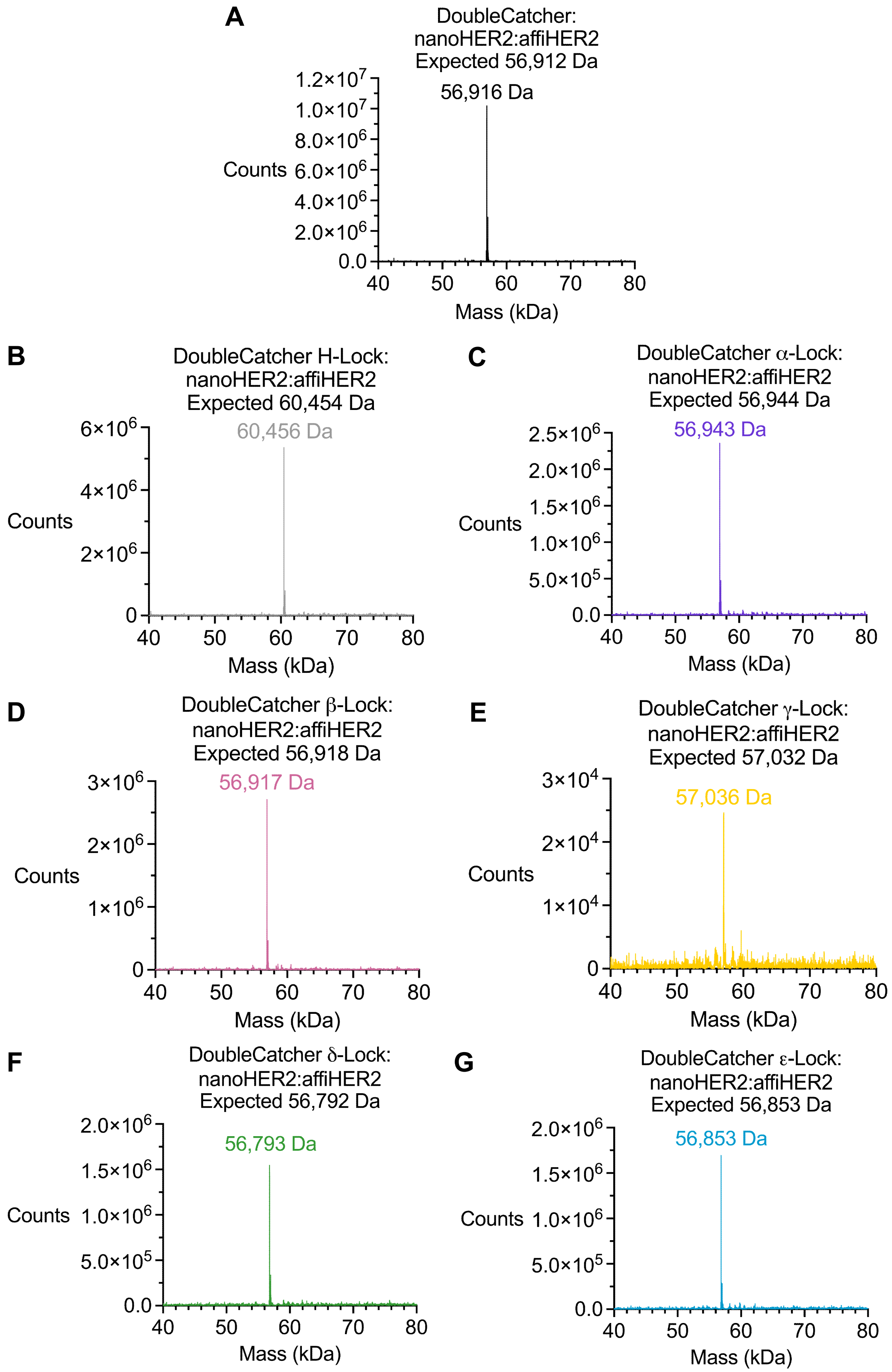

Supplementary Figure 8. **Heterodimerization of nanoHER2 and affiHER2 with the panel of DoubleCatcher variants is validated by mass spectrometry.** NanoHER2:affiHER2 bispecific binders were assembled using the full panel of DoubleCatcher variants. Purified bispecific molecules were analyzed by Electrospray Ionization Mass Spectrometry *(n* = 1 experiment per sample). Source data are provided as a Source Data file.
