# Supplementary material for: SpyMask enables combinatorial assembly of bispecific binders
Source: Nat Commun. 2024 Mar 16;15:2403. doi: 10.1038/s41467-024-46599-9 (PMC10944524; doi:10.1038/s41467-024-46599-9)
Supplement: Supplementary file 4 — Reporting Summary [file 41467_2024_46599_MOESM4_ESM.pdf]

nature portfolio

Corresponding author(s): Mark Howarth

Last updated by author(s): 11/02/2024

Reporting Summary

Nature Portfolio wishes to improve the reproducibility of the work that we publish. This form provides structure for consistency and transparency in reporting. For further information on Nature Portfolio policies, see our Editorial Policies and the Editorial Policy Checklist.

Statistics

For all statistical analyses, confirm that the following items are present in the figure legend, table legend, main text, or Methods section.

n/a	Confirmed	
☐	☒	The exact sample size (*n*) for each experimental group/condition, given as a discrete number and unit of measurement
☐	☒	A statement on whether measurements were taken from distinct samples or whether the same sample was measured repeatedly
☐	☒	The statistical test(s) used AND whether they are one- or two-sided<br>*Only common tests should be described solely by name; describe more complex techniques in the Methods section.*
☒	☐	A description of all covariates tested
☒	☐	A description of any assumptions or corrections, such as tests of normality and adjustment for multiple comparisons
☐	☒	A full description of the statistical parameters including central tendency (e.g. means) or other basic estimates (e.g. regression coefficient) AND variation (e.g. standard deviation) or associated estimates of uncertainty (e.g. confidence intervals)
☒	☐	For null hypothesis testing, the test statistic (e.g. *F*, *t*, *r*) with confidence intervals, effect sizes, degrees of freedom and *P* value noted<br>*Give P values as exact values whenever suitable.*
☒	☐	For Bayesian analysis, information on the choice of priors and Markov chain Monte Carlo settings
☒	☐	For hierarchical and complex designs, identification of the appropriate level for tests and full reporting of outcomes
☒	☐	Estimates of effect sizes (e.g. Cohen's *d*, Pearson's *r*), indicating how they were calculated

Our web collection on statistics for biologists contains articles on many of the points above.

Software and code

Policy information about availability of computer code

Data collection	SDS-PAGE data were acquired and analyzed using ImageLab version 6.1.0 (Bio-Rad) or iBright Analysis Software Versions 5.0.1 and 5.2.0 (Thermo Fisher).<br>Size-exclusion chromatography data was obtained using UNICORN version 6.4.1 SP2 (Cytiva).<br>Mass Photometry data were aquired using DiscoverMP v1.2.3 software (Refeyn Ltd).<br>Dynamic light scattering data were obtained using OmniSIZE version 3.0 software (Viscotek).<br>For ELISA assays, data were acquired using FLUOstar Omega version 5.10 R2.<br>For the metabolic activity assay (Resazurin), data were acquired using FLUOstar Omega version 5.10 R2.<br>ColabFold versions 1.3, 1.4 and 1.5.2 (Mirdita et al., 2022) were used for structural predictions of DoubleCatcher variants. Structures were validated with AlphaFold v2.3.1 (Jumper et al., 2021). AlphaFold-multimer was used for docking structure predictions of nanoHER2 to the HER2 extracellular domain (Evans et al. 2021).

Data analysis	Data visualization and statistical tests were performed using GraphPad Prism versions 9 and 10 (GraphPad Software).<br>Data visualization was also carried out using MATLAB R2023a (Mathworks).<br>Protein structures were visualized in PyMOL version 2.5.4 (Schrödinger) and vectors connecting two atoms within a protein structure were simulated in PyMOL using the modevectors tool (Law, 2020).<br>Amino acid sequences were analyzed using ExPASy ProtParam (Gasteiger et al, 2015, https://web.expasy.org/protparam/).<br>Signal peptide cleavage was predicted using SignalP 6.0 (Nielsen et al., 2019).<br>Dynamic light scattering data were processed using OmniSIZE 3.0 (Visotek).<br>Mass spectrometry data were analyzed using Mass Hunter Qualitative Analysis software B.07.00 (Agilent) or MassLynx software V4.2 SCN 971 (Waters).<br>Isopeptide bond reconstitution reaction data and metabolic activity assay data were analyzed using Microsoft Excel version 16.72.

For manuscripts utilizing custom algorithms or software that are central to the research but not yet described in published literature, software must be made available to editors and reviewers. We strongly encourage code deposition in a community repository (e.g. GitHub). See the Nature Portfolio guidelines for submitting code & software for further information.

Data

Policy information about availability of data

All manuscripts must include a data availability statement. This statement should provide the following information, where applicable:

- Accession codes, unique identifiers, or web links for publicly available datasets
- A description of any restrictions on data availability
- For clinical datasets or third party data, please ensure that the statement adheres to our policy

We have deposited plasmids in Genbank and the Addgene repository (https://www.addgene.org/Mark_Howarth/) with the unique identifiers listed: plasmids encode SnoopTag-AffiHER2-SpyTag (GenBank accession no. KU296975, Addgene plasmid ID 216280), SnoopTag-SpyTag-(AffiHER2)3 (GenBank Accession no. KU296976, Addgene plasmid ID 216281), SUMO-SpyTag003 (GenBank Accession no. PP341235, Addgene plasmid ID 216282), SpyCatcher003-TEVs-SpyTag003DA (GenBank Accession no. PP341217, Addgene plasmid ID 216283), DoubleCatcher (GenBank Accession no. PP341218, Addgene plasmid ID 216284), DoubleCatcher H-Lock (GenBank Accession no. PP341219, Addgene plasmid ID 216285), DoubleCatcher α-Lock (GenBank Accession no. PP341220, Addgene plasmid ID 216286), DoubleCatcher β-Lock (GenBank Accession no. PP341221, Addgene plasmid ID 216287), DoubleCatcher γ-Lock (GenBank Accession no. PP341222, Addgene plasmid ID 216288), DoubleCatcher δ-Lock (GenBank Accession no. PP341223, Addgene plasmid ID 216289), DoubleCatcher ε-Lock (GenBank Accession no. PP341224, Addgene plasmid ID 216290), MBPx-MBPx-SpyTag003 (GenBank Accession no. PP341236, Addgene plasmid ID 216291), SpyCatcher002-MBP (GenBank Accession no. PP341225, Addgene plasmid ID 216295), nanoHER2-SpyTag003 (GenBank Accession no. PP341234, Addgene plasmid ID 216312), and the heavy and light chain sequences of Tras-SpyTag003, Tras NoLink-SpyTag003, 39S-SpyTag003, MF3958-SpyTag003, H2-18-SpyTag003, and Pert-SpyTag003 [GenBank Accession nos. PP341226 (39S Heavy); PP341227 (39S Light); PP341228 (MF3958 Heavy); PP341229 (MF3958 Light); PP341230 (H2-18 Heavy); PP341231 (H2-18 Light); PP341232 (Pert Heavy); PP341233 (Pert Light), Addgene plasmid IDs: 216296 (39S Heavy); 216297 (39S Light); 216303 (MF3958 Heavy); 216304 (MF3958 Light); 216307 (H2-18 Heavy); 216308 (H2-18 Light); 216309 (Pert Heavy); 216310 (Pert Light)]. Requests for source data, resources, reagents, and further information should be made to the corresponding author, M.R.H.
All raw mass spectrometry data (from 33 runs) have been deposited to the ProteomeXchange via the PRIDE database with identifier PXD049393 http://proteomecentral.proteomexchange.org/cgi/GetDataset?ID=PXD049393. Source data are provided in the Source Data file.

Research involving human participants, their data, or biological material

Policy information about studies with human participants or human data. See also policy information about sex, gender (identity/presentation), and sexual orientation and race, ethnicity and racism.

Reporting on sex and gender	N/A
Reporting on race, ethnicity, or other socially relevant groupings	N/A
Population characteristics	N/A
Recruitment	N/A
Ethics oversight	N/A

Note that full information on the approval of the study protocol must also be provided in the manuscript.

Field-specific reporting

Please select the one below that is the best fit for your research. If you are not sure, read the appropriate sections before making your selection.

☒ Life sciences ☐ Behavioural & social sciences ☐ Ecological, evolutionary & environmental sciences

For a reference copy of the document with all sections, see nature.com/documents/nr-reporting-summary-flat.pdf

Life sciences study design

All studies must disclose on these points even when the disclosure is negative.

Sample size	No statistical methods were used to predetermine sample size.<br>Data in Fig. 1D; Fig. 2C, F; Fig. 4C–F; Fig. 6A, B; Supplementary Fig. 4C; Supplementary Fig. 5; and Supplementary Fig. 6D represent n = 3.<br>Data in Fig. 5F represent n = 10. For qualitative assessment, this is sufficient.
Data exclusions	No data recorded were excluded from analysis or data visualization.
Replication	Where applicable, experiments were repeated successfully at least once with similar or identical experimental setup. Representative SDS-PAGE (Fig. 1C, E; Fig. 2B, E; Supplementary Fig. 4A, B), observations were replicated successfully at least once with similar or identical conditions. ELISA assays (Supplementary Fig. 5; Supplementary Fig. 6D) were repeated at least once with similar or identical conditions and binding activity was replicated successfully. Heterodimer assembly by SpyMask and subsequent analyses by mass photometry and SDS-PAGE (Fig. 3 D, E) were repeated at least twice with similar results. For Fig. 4C, D and Fig. 6D, the anti-HER2 bispecific assemblies and subsequent metabolic activity assays were repeated independently at least twice in triplicate with similar results. For mass spectrometry experiments in Supplementary Fig. 2; Supplementary Fig. 3; and Supplementary Fig. 8, experiments were performed once per sample, and for Supplementary Fig. 7 experiments were confirmed once with independently-assembled DoubleCatcher heterodimers with identical results.
Randomization	Randomization was not used for any experiments in this study. No patient data or animal studies are contained within this manuscript.
Blinding	Blinding was not used for any experiments in this study. No patient data or animal studies are contained within this manuscript.

Reporting for specific materials, systems and methods

We require information from authors about some types of materials, experimental systems and methods used in many studies. Here, indicate whether each material, system or method listed is relevant to your study. If you are not sure if a list item applies to your research, read the appropriate section before selecting a response.

Materials & experimental systems

n/a	Involved in the study	
☐	☒	Antibodies
☐	☒	Eukaryotic cell lines
☒	☐	Palaeontology and archaeology
☒	☐	Animals and other organisms
☒	☐	Clinical data
☒	☐	Dual use research of concern
☒	☐	Plants

Methods

n/a	Involved in the study	
☒	☐	ChIP-seq
☒	☐	Flow cytometry
☒	☐	MRI-based neuroimaging

Antibodies

Antibodies used	Anti-HER2 Fabs Tras , Tras NoLink, 39S (Oganesyan et al., 2018), MF3958 (Geuijen et al., 2018), H2-18 (Hu et al., 2015), and Pert (Gennaro et al., 2014) were produced in-house.<br>Mouse Anti-Human IgG Fc Antibody conjugated to HRP was purchased from GenScript (cat. 50B4A9).
Validation	Anti-HER2 Fabs Tras, Tras NoLink, 39S, MF3958, H2-18, and Pert were produced in-house and have been verified to bind to distinct subdomains on the HER2 extracellular domain (ECD). In our study, the binding activity of each Fab fused to a SpyTag003 peptide on the heavy chain to recombinantly expressed HER2 ECD was validated by ELISAs. Crystal structures are available for Tras W102D, 39S, MF3958, H2-18, and Pert. Previous mutational studies validated the Tras epitope on HER2 to be subdomain IV (Phillips, GL et al., 2022), and the Pert epitope on HER2 to be subdomain II (Shin, JW et al., 2019). The 39S Fab epitope was validated to be subdomain II following the generation of human/mouse HER2 ECD chimeras, in which each subdomain of the human ECD was systematically replaced with its mouse counterpart (Oganesyan, V. et al., 2018). The MF3958 epitope subdomain I was determined by crystallography together with alanine scanning of the binding interface (Geuijen, CAW. et al., 2018). H2-18 was determined by crystallography (Hu, S. et al., 2015).<br><br>The Mouse Anti-Human IgG Fc-HRP conjugate antibody reacts with the Fc domain of human IgG but not with the Fab domain of human IgG, as verified by the manufacturer. The antibody has been successfully used for detection of human IgG Fc in applications including ELISA and Western Blot, and has been cited by at least 4 publications to date.

Eukaryotic cell lines

Policy information about cell lines and Sex and Gender in Research

Cell line source(s)	SKBR3 cells were from ATCC (HTB-30).<br>BT474 cells were from Cancer Research UK, Lincoln's Inn Fields.

April 2023

	Expi293F cells were obtained from Thermo Fisher (A14635).
Authentication	No further authentication of the cell lines was carried after out after acquisition from their suppliers.
Mycoplasma contamination	All the cell lines that were used in this study routinely tested negative for mycoplasma contamination by PCR.
Commonly misidentified lines (See ICLAC register)	There were no commonly misidentified lines used in this study.
